# Detection and validation of structural variations in bovine whole-genome sequence data

**DOI:** 10.1186/s12711-017-0286-5

**Published:** 2017-01-25

**Authors:** Long Chen, Amanda J. Chamberlain, Coralie M. Reich, Hans D. Daetwyler, Ben J. Hayes

**Affiliations:** 1 AgriBio, Centre for AgriBioscience, Biosciences Research, Department of Economic Development, Jobs, Transport and Resources, Bundoora, VIC Australia; 2 School of Applied Systems Biology, La Trobe University, Bundoora, VIC Australia

## Abstract

**Background:**

Several examples of structural variation (SV) affecting phenotypic traits have been reported in cattle. Currently the identification of SV from whole-genome sequence data (WGS) suffers from a high false positive rate. Our aim was to construct a high quality set of SV calls in cattle using WGS data. First, we tested two SV detection programs, Breakdancer and Pindel, and the overlap of these methods, on simulated sequence data to determine their precision and sensitivity. We then identified population SV from WGS of 252 Holstein and 64 Jersey bulls based on the overlapping calls from the two programs. In addition, we validated an overlapped SV set in 28 twice-sequenced Holstein individuals, and in another two validated sets (one for each breed) that were transmitted from sire to son. We also tested whether highly conserved gene sets across eukaryotes and recently expanded gene families in bovine were depleted and enriched, respectively, for SV.

**Results:**

In empirical WGS data, 17,518 SV covering 27.36 Mb were found in the Holstein population and 4285 SV covering 8.74 Mb in the Jersey population, of which 4.62 Mb of SV overlapped between Holsteins and Jerseys. A total of 11,534 candidate SV covering 5.64 Mb were validated in the 28 twice-sequenced individuals, while 3.49 and 0.67 Mb of SV were validated from Holstein and Jersey sire-son transmission, respectively. Only eight of 237 core eukaryotic genes had at least a 50-bp overlap with an SV from our validated sets, suggesting that conserved genes are depleted for SV (p < 0.05). In addition, we observed that recently expanded gene families were significantly more associated with SV than other genes. Long interspersed nuclear elements-1 were enriched for deletions when compared to the rest of the genome (p = 0.0035).

**Conclusions:**

We reported SV from 252 Holstein and 64 Jersey individuals. A considerable proportion of Jersey population SV (53.5%) were also found in Holstein. In contrast, about 76.90% sire-son transmission validated SV were present in Jerseys and Holsteins. The enrichment of SV in expanding gene families suggests that SV can be a source of genetic variation for evolution.

**Electronic supplementary material:**

The online version of this article (doi:10.1186/s12711-017-0286-5) contains supplementary material, which is available to authorized users.

## Background

There are several categories of genomic variations within a species. Single nucleotide polymorphisms (SNPs) are the most frequent and have been widely used in genome-wide association and genomic prediction. In the last decade, several studies have detected and verified the existence of large DNA segment mutations in the human genome [1] and many other species [2–4]. These segment mutations are now described as structural variations (SV), which refer to segments of 1 kilobases (kb) to several megabases (Mb) of deletions, duplications, inversions and translocations in a re-sequenced genome compared to a reference genome [5, 6]. Copy number variations (CNV) are a subset of structural variations including deletions and duplications. As sequencing technology has improved, the resolution of the detection of structural variations has also improved, thus, smaller SV events can now be identified [7].

In humans, the first paper that reported widespread CNV included evidence that this type of variation plays a significant role in population heritable variation [7]. SV have also been shown to be significantly associated with complex diseases, especially schizophrenia [8], rheumatoid arthritis [9] and systemic lupus erythematosus [10]. In cattle, several studies have indicated that SV that span gene coding regions can affect a range of traits [11]. In Angus cattle, 297 CNV were found to be associated with parasite resistance or susceptibility and to overlap with 437 genes enriched for immune function [12]. Recently, a 660-kb deletion was found to be associated with fertility and milk production in Nordic red cattle [13]. In addition, an 80-kb duplication on BTA1 (BTA for *Bos taurus* chromosome) was shown to be associated with the polled phenotype in Friesian origin cattle, and a 202-bp complex insertion-deletion on BTA21 with polled in Celtic origin cattle [14, 15].

It is plausible that SV are responsible for variation in many complex traits in cattle, including milk production, fertility, and other traits. To test this hypothesis on a genome-wide scale, a genome-wide catalogue of SV in bovine populations must first be developed. Various types of genomic data can be used to detect SV. SNP array data has enabled a rapid high-throughput approach for identifying genetic variants, using signal intensity information. The CNV detection program PennCNV implements a hidden Markov model (HMM) to detect loss or gain of CNV status from SNP arrays [16]. The results are relatively reliable and thus this program has been used in many CNV studies. However, due to limited SNP array density and the high minor allele frequency of these SNPs, identification of smaller and rare CNV, and determination of exact breakpoints are limited. In addition, SNP chip methods cannot capture balanced SV including inversions and translocations.

Massive parallel sequencing or whole-genome sequence data (WGS) is becoming increasingly cost-effective in genotyping studies. WGS data can potentially be used to recover the whole spectrum of SV. Paired-end mapping (PEM), split read (SR), read depth (RD), and de novo assembly are the current four basic strategies used to detect SV from sequence data [17]. Breakdancer [18] uses PEM information, while Pindel [19] uses SR information. Both strategies have been applied to cancer genome projects and the 1000 genomes project in humans [20, 21]. No single method is able to detect the entire spectrum of SV events, so studies usually combine two or three strategies to achieve better results depending on the research targets [17].

Here, we first tested the precision and sensitivity of Breakdancer, Pindel, and a combined strategy to detect SV in simulated bovine WGS data. To expand the catalogue of SV in cattle, with a very high level of confidence, we then detected SV in WGS data from Holstein and Jersey populations using a combination of Breakdancer and Pindel, as well as two novel validation strategies including sire-son transmission and evidence from animals sequenced several times. The overlap of validated SV with those detected by PennCNV was also investigated. Furthermore, we also tested the hypothesis that gene regions that are highly conserved between species should have fewer SV than in less conserved gene regions, while recently expanded gene families in bovine could have more SV than other regions.

## Methods

### SV programs

We used Breakdancer (version 1.4.4) and Pindel (version 0.2.5a3) programs to detect SV. Since Pindel is not applicable for intra-chromosome duplications (i.e. CTX in Breakdancer), we only used deletions (DEL), insertions (INS), inversions (INV) and tandem duplications (DUP) for comparisons between the two programs.

### Simulated dataset

To gain insight into the power and precision of Pindel and Breakdancer to detect SV, we used a simulated dataset that was generated from simulated short reads based on BTA29 extracted from the UMD3.1 bovine genome reference assembly. Rearranged chromosomes with 300 randomly inserted SV (100 SV each for deletions, inversions and duplications) were generated with the R package RSVSim [22]. Simulation of short reads was achieved by using *wgsim* [23].

We considered several factors that may influence the performance of SV detection: homozygous or heterozygous SV; the sequence base error rate; SV in low complexity regions, i.e. repetitive regions of the genome; and the percentage of SNPs within the flanking regions of the breakpoints of a SV. We simulated six scenarios that considered each of these factors (Table 1).

**Table 1:** Parameters for the simulation scenarios

Simulation set	HOM	HET	Base error rate	Repetitive	SNP% in SV	Mix
HOM/HET	HOM	HET	HOM	HOM	HOM	HET
SNP% in SV	0	0	0	0	0.01–0.25	0.01
REP region	0	0	0	100%	0	50%
Number of SV	100 × 3	100 × 3	100 × 3	100 × 3	100 × 3	100 × 3
Insert size	500	500	500	500	500	500
SD of insert size	50	50	50	50	50	50
Base error rate	0.01	0.01	0.001–0.025	0.01	0.01	0.01
SNP rate	0.01	0.01	0.01	0.01	0.01	0.008
Indel rate	0.01	0.01	0.01	0.01	0.01	0.01

HOM/HET represents homozygous and heterozygous SV, respectively; SNP% in SV represents the percentage of SNPs that occur in a SV region; REP region represents the percentage of SV that fall in repetitive regions (LINE regions). One hundred each for deletions, inversions and tandem duplications were simulated under each simulation. Default insert size and standard deviation of insert size of 500 and 50, respectively, were used; SNP rate is the overall SNP percentage that exists across the whole cattle genome

For each simulation set, the insert size was set at 500 bp with a 50-bp standard deviation and the indel rate was set at 0.01. In the first two scenarios, in which all SV were either homozygous or heterozygous (HOM, HET), no SV fell into repetitive regions and no SNPs appeared within SV events. For HOM SV, 100 randomly distributed deletions, inversions and duplications, respectively, were inserted into BTA29 to form a rearranged BTA29. Fifty replicate sets of rearranged BTA29 were generated and, based on each rearranged chromosome, 50 replicates of short reads were then simulated with ~tenfold sequence coverage. For HET SV, both the number of SV and coverage per SV were halved to 50 and 5, respectively. One hundred rearranged BTA29 chromosomes were then generated and reads that were simulated from two different rearranged BTA29 chromosomes were pooled together to simulate heterozygous SV and tenfold coverage (see Additional file 1: Figure S1). The third scenario varied the base error rate from 0.001 to 0.025, while for other scenarios the base error rate was set at 0.01. A fourth scenario investigated an extreme case where all SV were in repetitive regions of the genome, i.e. 300 repetitive regions were randomly selected based on the UCSC genome browser database and simulated SV were inserted only into those chosen regions. A fifth scenario considered a range of rates (0.01 to 0.25) of SNPs that occur in SV (number of SNPs divided by the total SV length in bp). Finally, in an attempt to more closely match the real genome structure, we incorporated all the above factors together in one scenario (MIX): all SV were heterozygous with a proportion of SNPs within SV of 0.01, and half of these SV were in repetitive regions. This also included a SNP polymorphism rate calculated from the Holstein animals in the 1000 Bull Genomes project (0.008 per locus).

The precision and sensitivity of SV calls of Breakdancer and Pindel were then compared. Precision was defined as the number of true positives divided by the number of total calls made by the program. Sensitivity equalled the number of true positives divided by the number of actual variants in the simulations. A true positive SV was defined as an SV call that was detected by the program with at least 50% overlap with a simulated SV.

### Animal samples

Many of the sequences used in this study were described and published by Daetwyler et al. [24]. Two hundred and eighty Holstein animals (of which 28 were sequenced twice) and 64 Jersey animals were sequenced using the Illumina sequencing technology (see Additional file 2). Information on coverage and insert size is summarised in Table 2. All sequence reads were then aligned to the reference assembly UMD 3.1 with the Burrows-Wheeler Aligner (BWA) [25].

**Table 2:** Genome coverage read depth and insert size of SV for the WGS datasets

Population	Number	Coverage			Insert size		
		Min	Mean	Max	Min	Mean	Max
Holstein	308	3.21	10.81	44.53	250	347.6656	514
Jersey	64	3.45	10.92	25.68	250	364.5469	502

### Sequence population SV calls

We pooled the Holstein (not including twice-sequenced individuals) and Jersey populations and investigated the SV distribution for the two breeds. For each population, we first ran Breakdancer and Pindel to generate raw SV calls for each SV type (deletion, insertion, inversion and duplication). Picard tools “CollectInsertSizeMetrics.jar” was used to calculate the mean insert size for each bam file. The default parameters were used for both programs. However, we enforced an initial threshold of a minimum of four supporting read pairs and observations of a SV in at least two individuals to classify higher quality SV. SV that spanned chromosome gaps in the reference assembly were also filtered out. We applied an overlap size of at least 25 bp between the two programs to retain smaller SV. We only included SV that were detected by both Breakdancer and Pindel and considered that these overlapping SV had a higher confidence level. The overlapping SV sets were named POP_HOL and POP_JER for Holstein and Jersey populations, respectively. We performed a *t* test to check whether the overlap of the proportion of genome covered with SV by the two programs was significantly different than expected by chance. For each chromosome, the expected overlap proportion was calculated as the proportion of the genome covered by Breakdancer SV calls multiplied by the proportion of Pindel SV calls. These were contrasted with the actual overlapping genome region across 30 chromosomes (n = 30) and for each SV type separately. A flowchart of the pipeline is in Additional file 1: Figure S2.

### Validation of SV

In the Holstein population, 28 individuals were sequenced twice. In theory, for each individual the two independent sequences should support exactly the same SV calls. However, due to random distribution of sequence reads, assembly errors and different depths of coverage, the two generated sequences are not identical, and, thus, programs report different SV. We generated a set of high confidence SV by only reporting the SV that were detected in both sequences (TWICE_SEQ). Furthermore, we also combined the bam files from 21 of the 28 twice-sequenced individuals for which insert sizes did not differ by more than 5 bp (MERGE). The number of SV from the MERGE set was then checked for overlap with the TWICE_SEQ set.

In addition, there were 68 Holstein and 33 Jersey sire-son pairs in the dataset, of which some sons shared the same sire. As SV should be inherited (100% of the time if the sire is homozygous and 50% of the time if the sire is heterozygous for an SV), we only reported SV that were inherited from sire to offspring in at least one pair. The resulting SV sets were named as FAM_HOL and FAM_JER for Holstein and Jersey sire-son pairs, respectively. The two validated sets were further compared between each other and with SV called from SNP chip data. The pipeline for generating validated sets is in Additional file 1: Figures S3 and S4.

### Detection of CNV from SNP chip genotype data

One hundred and twenty-eight Holstein and 170 Jersey cattle (the majority of these animals were in the sequenced set as well) were genotyped with the bovine 800 K HD SNP chip. Their converted Log R ratios (LRR) and B allele frequencies (BAF) were used to call SV with PennCNV. Individuals with a standard deviation of LRR higher than 0.35 and BAF higher than 0.2 were discarded, as suggested by Wang et al. [16]. One hundred and twenty-five Holstein and 166 Jersey were retained after this filtering step. The genomic content (GC) model, which incorporates information on GC percentage around each SNP, was used to improve CNV calls. SNP chip methods cannot detect inversions and therefore we excluded inversion events when comparing PennCNV called CNV to validated sets from sequence data.

### Conserved genes

To test the hypothesis that SV and CNV are less likely in genes that are highly conserved across species, 248 core eukaryotic genes (CEG) were selected [26] that were likely to be present in a small number of paralogs in a wide range of species. We downloaded the protein file (fasta format) and used the BLAST program [27] to detect the most similar proteins and genes in cattle. The search results were further converted into coding nucleotides in bed format with chromosome, strand, start and end position that can be overlapped with our validated SV sets. We required that a minimum of 50 bp of the gene overlapped with the validated SV for the gene to be reported. A Chi squared test (χ^*2*^ = ∑ (E – O)^2^/E, where E is the expected number of genes assuming that conserved genes and SV are independent and O is the observed number of genes (i.e. conserved or non-conserved, and overlapping or non-overlapping with SV), was performed to test whether these conserved genes contain less SV than expected by chance, with all the other reference genes across the genome downloaded from the UCSC genome browser.

### Structural variants in expanded gene families

Gene families are sets of genes that originated from a common ancestor and formed by gene duplication [28]. CNV are considered as a major source of variation for gene family evolution and expansion [29]. First, we searched the literature for expanded gene families in the bovine genome and found five reported expanded gene families: *pregnancy*-*associated glycoprotein* [30], *prolactin* (*PRP*) [31], bovine *beta*-*defensin* (*DEFB*) [32], *cathelicidin* (*CATHL*) [33] and *NK*-*lysin* [34], with the *DEFB* family containing four clusters (clusters A to D referring to BTA8, 13, 23 and 27, respectively). We retrieved the coding sequences for these genes from the UCSC genome browser and searched for SV in these regions. We also performed Chi squared tests for these gene families to test whether they contain more SV than expected by chance, as for the conserved genes above.

Some gene family expansions are due to retrotransposons [35]. Long interspersed nuclear elements (LINEs) are abundant retrotransposons in mammals. Although 99.9% of LINEs are not able to mobilize [36], one subgroup of LINEs, L1, is the only element that is still active in mammalian genomes [37]. L1-mediated retrotransposon events can lead to various structural variations and diseases [38]. We hypothesised that L1 elements were involved with more SV than the other regions that were not under selective constraint (i.e. outside of exons). The L1 regions were retrieved from the UCSC genome browser and were compared with the validated SV sets to check how many SV fall into these regions. We applied a t test to compare the proportion of regions that are affected by SV between the L1 and other regions (excluding exons and L1) in the genome.

## Results

### Simulated data

In the simulated data, both Breakdancer and Pindel detected similar numbers of homozygous duplications (see Additional file 3: Table S2) and a high proportion of these were true positives. Breakdancer reported more deletions and many more inversions than Pindel. Breakdancer detected a similar proportion of heterozygous and homozygous SV, whereas Pindel detected fewer heterozygous SV than homozygous SV, and the proportion of deletions detected was almost halved for heterozygous SV (see Additional file 3: Table S2).

**Fig. 1:**
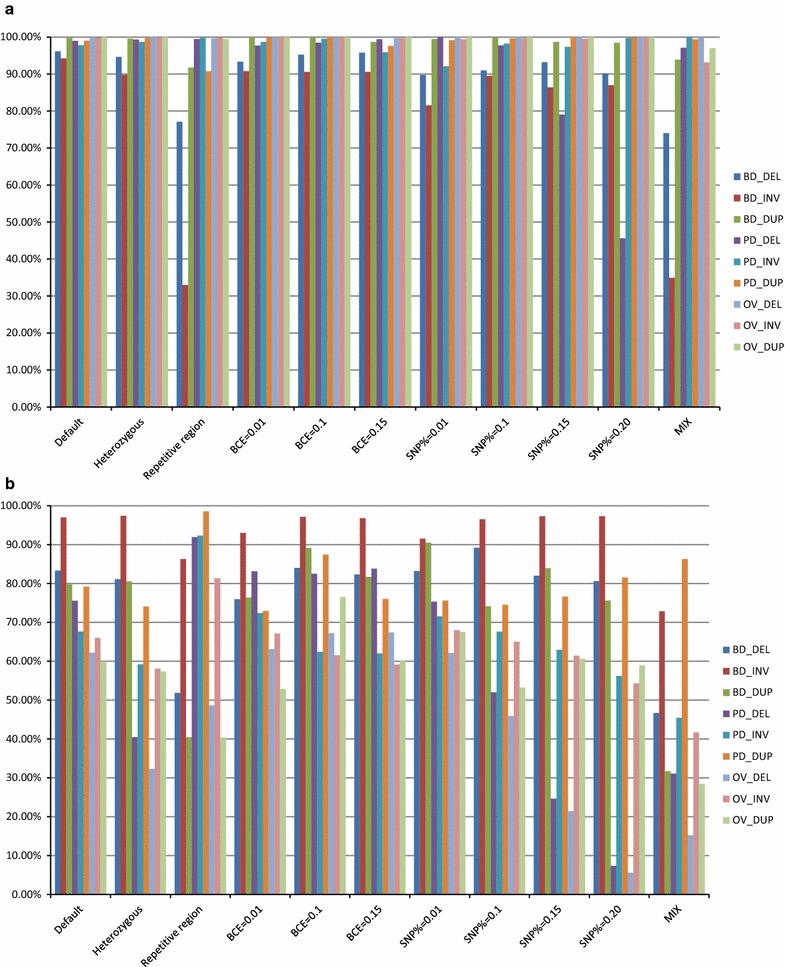
Precision and sensitivity of Breakdancer, Pindel and overlap methods for the detection of structural variations in different simulation scenarios. **a** Precision of each method; **b** sensitivity of each method; *BD* Breakdancer, *PD* Pindel, *OV* overlap method, *DEL* deletions, *INV* inversions, *DUP* duplications. *BCE* base calling error rate, *MIX* mix scenario with SNP% = 0.01, SNP rate = 0.008 and half of the SV falling into repetitive regions. Precision is defined as the average number of true positives divided by the average number of total calls made by each program. Sensitivity is defined as the average number of true positives divided by the average number of actual variants in the simulations

Both Breakdancer and Pindel performed well in terms of precision (true positives divided by total calls) for both homozygous and heterozygous SV (Fig. 1). The lowest precision 89.8% was found with Breakdancer for the identification of inversions. The overlapped sets from Breakdancer and Pindel improved the precision by up to 10% when compared to each program separately. Sensitivity (true positives/total variants in the simulation) for Breakdancer and Pindel differed between homozygous and heterozygous SV. While both methods captured around 80% of the simulated homozygous deletions and duplications, Breakdancer identified 87% inversions while Pindel only detected 58%. For heterozygous SV, the sensitivity of Breakdancer remained at a similar level for each type, while the sensitivity of Pindel was reduced by 35% for deletions, 8.5% for inversions and 5.15% for duplications, respectively.

We attempted to identify situations in which the SV detection programs performed less well than in the ideal conditions simulated above.

Figure 1 shows the precision and sensitivity for each simulation scenario. When data were simulated with higher per base call error rates, we did not observe large decreases in precision. However, when low-complexity regions were simulated, the average precision of SV detection dropped by 19.04, 61.27 and 7.98% when detecting deletions, inversions and duplications, respectively, by Breakdancer, while Pindel had a 8% drop in precision for detecting duplications (see Additional file 3: Table S3). Another more extreme simulation investigated a per locus SNP rate in SV higher than 0.2, in which the precision for detecting deletions with Pindel dramatically fell to 46%. In the MIX scenario (SNP% = 0.01, SNP rate = 0.008 and 50% SV in repetitive regions), we observed a relatively low precision for deletions and a much lower precision for inversions when using Breakdancer. While the precision and sensitivity of SV detection in the simulated data were higher than those that are likely in real studies (in spite of our best efforts to simulate a realistic sequence), the results do demonstrate that the combination of the two programs (Breakdancer and Pindel) yields higher precision, but lower sensitivities, than either program alone under different situations. Therefore, we concluded that the combined approach yielded higher confidence SV calls, which supported our validation strategy in empirical data.

### Population SV calls

**Fig. 2:**
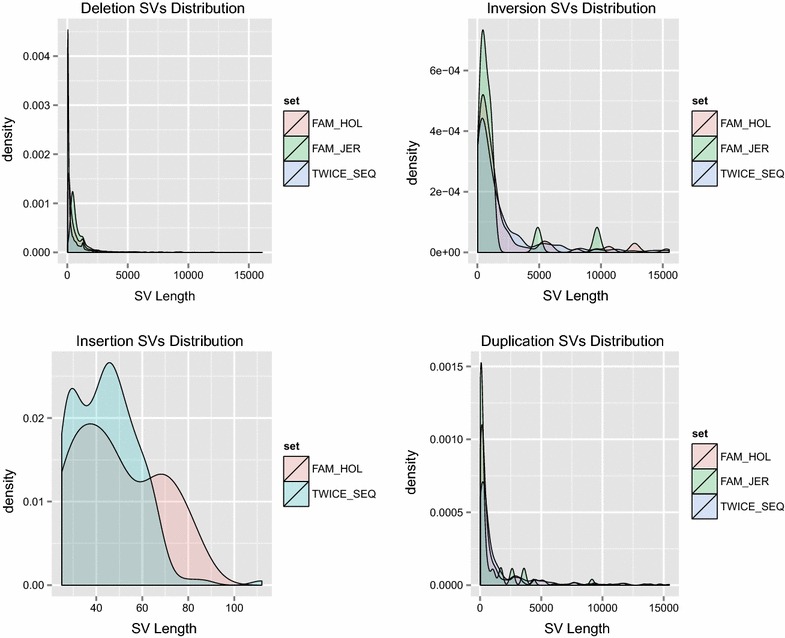
Size distribution of four types of structural variations in validation datasets (SV in twice-sequenced and Holstein and Jersey sire-son transmission sets). The x axis represents the length of SV; the y axis represents the frequency of SV for each length; the *pink area* represents the Holstein sire-son transmission validated set; the *green area* represents the Jersey sire-son transmission validated set; the *blue area* represents the twice-sequenced validated set

Breakdancer and Pindel reported a different number of SV for the Holstein and Jersey WGS data (Table 3; Additional file 3: Table S4). The SV that overlapped between Breakdancer and Pindel ranged from 0.55 to 28% of the SV detected by Breakdancer, and from 1.69 to 11.21% of the SV detected by Pindel. Therefore, the overlapping sets dramatically shrunk the original number of SV. However, there was much more overlap in the SV detected by the two programs than expected by chance (t test, p < 5 × 10^−9^). Thus, thereafter, the results are only presented for the overlapped or validated sets. Overall, Holstein had more SV calls than Jersey, which is likely due to a larger sample size for Holstein. The size of SV ranged from 25 to 44,412 bp, where the median length of deletions, insertions, inversions and duplications for Holstein was 1123 bp, 72 bp, 2533 bp and 857 bp and for Jersey was 1152 bp, no insertions detected, 1337 bp and 1014 bp, respectively (Fig. 2). Table 4 shows the total covered length of SV shared by the two populations. A total of 27.36 Mb and 8.64 Mb of SV were detected in the Holstein and Jersey populations, respectively. 53.5% of the SV found for the Jersey population (4.62 Mb) were also shared by the Holstein population.

**Table 3:** Number and length of genome regions covered by SV detected in the Holstein and Jersey sets by Breakdancer and Pindel

RAW_SV_output	SV counts				SV covered region (Mb)			
SV Set	DEL	INS	INV	DUP	DEL	INS	INV	DUP
POP_HOL_Breakdancer	2,124,795	2,047,019	46,975	28,745	116.97	115.47	118.82	15.69
POP_HOL_Pindel	51,302	85,946	457,575	21,888	144.96	6.35	269.69	84.86
POP_JER_Breakdancer	412,830	498,257	4397	4502	31.56	32.77	13.30	7.98
POP_JER_Pindel	37,717	47,234	63,683	20,889	46.58	3.38	62.28	27.53

**Table 4:** Number and length of genome regions covered by SV detected in the Holstein and Jersey sets and the three validated sets, and in the overlapped set between Holstein and Jersey

Final SV output set	SV counts				SV covered region (Mb)			
SV Set	DEL	INS	INV	DUP	DEL	INS	INV	DUP
POP_HOL	4037	7679	3623	2179	8.4889	0.6334	13.8377	4.3995
POP_JER	2679	0*	415	1191	5.2239	0.0000*	1.0497	2.3675
Overlap between POP_HOL and POP_JER	1533	0	69	601	3.1790	0.0000	0.2188	1.2270
TWICE_SEQ	10,893	174	200	267	4.8495	0.0077	0.3882	0.3934
FAM_HOL	4230	24	106	258	2.9639	0.0012	0.2057	0.3173
FAM_JER	619	0*	17	58	0.5944	0.0000*	0.0240	0.0466
Overlap between FAM_HOL and FAM_JER	509	0	14	27	0.4704	0.0000	0.0185	0.0225

* No insertions were found for the Jersey population

### Validated SV calls

Given the rates of false positives from both Breakdancer and Pindel, only validated SV should be considered further. Based on the overlapped calls from Breakdancer and Pindel, we generated three sets of validated SV calls: twice-sequenced Holstein animals (TWICE_SEQ), and a set of Holstein (FAM_HOL) as well as a set of Jersey (FAM_JER) SV that, as we demonstrated, had Mendelian inheritance (passed from sire to son). Summary statistics for these sets are in Table 4. In the TWICE_SEQ set, most of the SV were detected in both whole-genome sequences, however, some individuals showed large discrepancies between the two sequences (see Additional file 3: Table S5). A total of 5.64 Mb of SV were validated in TWICE_SEQ, while 3.49 Mb and 0.67 Mb of SV were validated in FAM_HOL and FAM_JER. When comparing the SV between FAM sets, 76.90% of the SV that were confirmed in Jersey were also confirmed in Holstein sire-son pairs. Eighty-two percent of the SV were found in both the TWICE_SEQ and the FAM_HOL set. This result suggested fewer false positives and thus higher confidence SV with either validated set compared to non-validated population calls. Figure 2 demonstrates that the size distribution of SV was similar across validated sets. Most deletions and insertions were less than 100 bp (as the maximum size of an insertion is determined by insert size); the size of a large number of inversions was around 900 bp, while that of duplications was around 350 bp. For inversions in FAM_JER, there were two small peaks at 5 kb and 10 kb, respectively. When examining the sires with multiple sons, about 80 kb of the deletions and 90 kb of the duplications on BTA1 were shared in Holstein and 27 kb of the inversions on BTA11 and 16 kb of the duplications on BTA14 were shared in Jersey, which suggests that these regions could be common CNV regions to both breeds.

We calculated the percentage of SV from the twice-sequenced set that were reported by both the overlapping (TWICE_SEQ) and merging (MERGE) of BAM method. Most of the reported SV (74.2% across 21 animals) were detected in both sequenced sets (Table 5). The relatively high concordance rate further supports the merit of our validations.

**Table 5:** Comparison of the TWICE_SEQ and MERGE sets for 21 twice-sequenced individuals

Animal ID	MERGE	OVERLAP	SHARE	Overlap%	Coverage
HOLFRAM268	845	27	15	55.56	21.32
HOLFRAM266	1896	1635	1041	63.67	14.25
HOLNLDM273	1796	1913	1176	65.48	15.34
HOLNLDM270	1677	2069	1136	67.74	15.51
HOLDNKM259	1682	2001	1146	68.13	15.74
HOLUSAM277	2219	2398	1524	68.68	17.62
HOLNLDM272	2039	2520	1410	69.15	16.91
HOLDEUM255	1881	2168	1341	71.29	17.02
HOLUSAM280	2000	2200	1440	72.00	17.55
HOLNLDM274	690	1585	497	72.03	14.78
HOLDNKM262	980	2537	714	72.86	17.4
HOLDNKM261	1969	1867	1361	72.90	15.84
HOLUSAM278	1011	3067	761	75.27	18.96
HOLDNKM260	2557	1305	986	75.56	16.93
HOLDEUM256	1059	2730	806	76.11	17.48
HOLSWEM275	1214	2882	926	76.28	18.86
HOLUSAM279	1331	2581	1036	77.84	16.75
HOLDNKM263	1159	2697	916	79.03	17.02
HOLDEUM257	1356	3626	1087	80.16	21.53
HOLCANM253	1600	257	255	99.22	42.71
HOLUSAM276	845	132	131	99.24	16.89

MERGE and OVERLAP represent the counts of SV that were observed by using the merge and overlap method, respectively. SHARE represents the counts of SV that were found by both methods. The overlap percentage is equal to SHARE counts divided by the smaller number found in the merge and overlap method. Coverage is the sum of the coverages for each twice-sequenced individual

The 800 K SNP chip data results from PennCNV indicated a total of 2224 CNV covering 250.5 Mb in Holstein (227 Mb deletions and 23.3 Mb insertions) and 2976 CNV covering 357.4 Mb in Jersey (333 Mb deletions and 24.3 Mb insertions). Since the resolution of the SNP platform is limited, PennCNV cannot detect smaller SV. Therefore, we only compared the PennCNV calls with SV that were larger than 5 kb and detected from the sequence data. As a result, 12.33% of the deletions and 11.59% of the duplications in the validated sets were also found in the Holstein PennCNV analysis, while 14.95% of the deletions and none of the duplications overlapped in Jersey.

The location and length of all validated SV call sets that were pooled and merged across all validations are in Additional file 4: Table S6. Note that INV calls should be treated with some caution, since false INV events can arise from tandem duplications. For example, if one read mapped to one unit and its paired read mapped to another highly similar neighbouring unit, this can be difficult to identify.

### Test on conserved genes

A list of all RefSeq genes that overlap with SV is summarised in Additional file 5. Of the 248 genes that are highly conserved across eukaryotes, 237 unique bovine genes were fully mapped to the core gene set [21]. After overlapping with our validated SV sets, eight different genes in the core eukaryotic genes (CEG) were involved with SV (Table 6), among which the *DENR* gene on BTA29 was completely encompassed by deletion events.

**Table 6:** Structural variation found in a set of genes that are highly conserved across eukaryotes

Gene name	Chromosome	Start bp	End bp	SV type	Dataset
*PIGK*	Chr3	67,687,801	67,824,633	DEL	TWICE_SEQ
*ELP3*	Chr8	10,456,053	10,576,397	DEL	TWICE_SEQ
*GTF2H2*	Chr20	9,851,676	10,148,631	DEL	TWICE_SEQ
*SKIV2L2*	Chr20	23,727,320	23,853,125	DEL	TWICE_SEQ
*ETFA*	Chr21	31,993,936	32,063,870	DEL	TWICE_SEQ
*IMP3*	Chr21	33,646,396	33,647,527	DEL	TWICE_SEQ
*DENR*	Chr29	7,723,699	7,725,004	DEL	TWICE_SEQ
*IARS*	Chr8	85,268,883	85,350,117	INV	TWICE_SEQ
*ETFA*	Chr21	31,993,936	32,063,870	DEL	FAM_HOL
*IMP3*	Chr21	33,646,396	33,647,066	DEL	FAM_HOL
*IARS*	Chr8	85,268,883	85,350,117	INV	FAM_HOL

No CEG were found in the Jersey family set. Compared to all other reference sequence genes, the Chi squared test indicated that conserved genes regions contained less structural variants than the other genes in the genome at p < 0.05 significant level (p = 0.025 for both TWICE_SEQ and FAM_HOL). The Chi square table for TWICE_SEQ and FAM_HOL sets are in Table 7.

**Table 7:** Chi squares and p values for the test on conserved genes

	Conserved	Non-conserved	Chi square	p value
TWICE_SEQ_SV				
SV	8	965	5.0155	0.025
not_SV	229	12555		
Total	237	13,520		
FAM_HOL				
SV	3	565	4.9937	0.02544
not_SV	234	12,955		
Total	237	13,520		

### Expanded gene families

The genes involved in the expanded gene families are described in Table 8. We found that nine of the 34 *DEFB* genes overlapped with our SV sets, mainly in cluster B and cluster D (0/4 in cluster A, 4/17 in cluster B, 1/5 in cluster C and 4/8 in cluster D). For the other gene families, SV in *CATHL*2 were found in both Holstein and Jersey populations; SV in *PAG16*, *PAG18*, *PRP6* and *PRP11* were found in the Holstein population and twice-sequenced sets. No SV were found in the *NK*-*lysin* gene family.

**Table 8:** Expanded gene families in the bovine genome with structural variations

Gene	Chr	Start bp	End bp	SV type	SV sets
DEFB122*	Chr13	61,561,981	61,578,126	DEL	POP_HOL;TWICE_SEQ;POP_JER
*DEFB122*	Chr13	61,561,981	61,578,126	INS	POP_HOL
*DEFB122A*	Chr13	61,562,053	61,566,096	DEL	POP_HOL;POP_JER
*DEFB122A*	Chr13	61,562,053	61,566,096	INS	POP_HOL
*DEFB125*	Chr13	61,371,090	61,377,521	INV	POP_HOL
*DEFB125A*	Chr13	61,391,541	61,402,435	INV	POP_HOL
*DEFB112*	Chr23	22,381,986	22,387,950	DEL	TWICE_SEQ
*DEFB*	Chr27	5,457,175	5,465,032	INS	POP_HOL
*DEFB1*	Chr27	5,483,406	5,539,158	INS	POP_HOL
*DEFB1*	Chr27	5,448,917	5,465,074	INS	POP_HOL
DEFB1*	Chr27	6,223,483	6,225,131	DEL	FAM_HOL;TWICE_SEQ;POP_JER
*DEFB52*	Chr27	5,134,073	5,276,254	DUP	POP_JER
*DEFB52*	Chr27	5,134,073	5,276,254	INS	POP_HOL
*DEFB52*	Chr27	5,134,073	5,276,254	DEL	POP_JER
*DEFB33*	Chr27	5,245,806	5,351,104	INS	POP_HOL
CATHL2*	Chr22	52,189,557	52,191,061	DEL	POP_HOL;POP_JER
*PAG16*	Chr29	38,952,100	39,189,606	DEL	TWICE_SEQ
*PAG16*	Chr29	38,952,100	39,189,606	INS	POP_HOL
*PAG16*	Chr29	38,952,100	39,189,606	INV	POP_HOL
*PAG18*	Chr29	38,428,102	38,437,106	DEL	TWICE_SEQ
*LOC751562*	Chr23	34,386,963	34,491,996	DEL	FAM_HOL;POP_HOL;TWICE_SEQ
*LOC751562*	Chr23	34,386,963	34,491,996	DUP	POP_HOL;POP_JER
*PRP6*	Chr23	34,479,662	34,491,996	DEL	POP_HOL;TWICE_SEQ

* Genes that are completely spanned by SV

To compare whether these gene families were enriched for SV (more than expected by chance), we further selected genes that had more than 50% of their sequence involved in SV and performed a Chi squared test. Three genes (*DEFB122* from cluster B, *DEFB1* from cluster D and *CATHL2*) were completely encompassed by SV. The Chi squared test results indicated that cluster B and D within the *beta*-*defensin* gene contained more SV than expected by chance (Table 9).

**Table 9:** Chi squares and p values for expanded gene families analysis

	*DEFB* cluster B	Other refseq genes	Chi square	p value
SV	4	969	7.0135	0.00809
Non-SV	13	12,771		
Total	17	13,740		

	*DEFB* cluster D	Other refseq genes	Chi square	p value
SV	4	969	22.4428	0.000002
Non-SV	4	12,780		
Total	8	13,749		

The Line 1 (L1) regions were retrieved from the UCSC genome browser. L1 regions represented 339.76 Mb of the bovine genome. The proportions of the L1 and other regions that were concordant with SV and the level of enrichment higher than expected by chance are illustrated in Table 10. While the three other types of SV had similar proportions in the L1 and other regions, the L1 regions were enriched with deletions, and this was consistent in all three validated sets (Table 10).

**Table 10:** Proportion of structural variants in LINE regions, compared with the genome as a whole and other regions

Sample set	Non-L1_exon	L1	Fold_change	t test p value
Deletions				0.003538
FAM_HOL	0.000805	0.003322	4.124097	
FAM_JER	0.000139	0.000818	5.893840	
POP_HOL	0.002868	0.005747	2.003659	
POP_JER	0.001654	0.004282	2.588642	
VAL_SV	0.001384	0.004992	3.608023	
Insertions				0.185507
FAM_HOL	0.000001	0.000000	0.000000	
FAM_JER	0.000000	0.000000	0.000000	
POP_HOL	0.000249	0.000196	0.787844	
POP_JER	0.000000	0.000000	0.000000	
VAL_SV	0.000003	0.000003	1.080158	
Inversions				0.260667
FAM_HOL	0.000084	0.000040	0.479628	
FAM_JER	0.000010	0.000005	0.484786	
POP_HOL	0.005262	0.005435	1.032979	
POP_JER	0.000395	0.000437	1.106285	
VAL_SV	0.000152	0.000123	0.810684	
Duplications				0.082899
FAM_HOL	0.000122	0.000115	0.945850	
FAM_JER	0.000016	0.000033	2.100240	
POP_HOL	0.001611	0.002144	1.331104	
POP_JER	0.000828	0.001416	1.710535	
VAL_SV	0.000148	0.000164	1.103813	

Fold change is equal to the percentage of the genome that harbors SV in the L1 regions divided by the percentage of the genome that harbors SV in the other regions

## Discussion

First, we detected bovine SV using a stringent pipeline that accepted SV only when there was support from both split read and pair end mapping information. In addition, this overlapping set obtained from combining Breakdancer and Pindel was subjected to two further validations, i.e. SV had to occur (1) in both aligned sequences of animals that were independently sequenced twice, and (2) in inherited sire-son pairs. We found that a large number of SV was shared by Holstein and Jersey populations (53.5% of the SV found in the Jersey genome were also found in the Holstein genome), which suggests that there are some common SV among cattle breeds. Ideally, we should have performed molecular validations to further test our approach. However, this was beyond the scope of our study.

The proportion of the genome displaying SV, which we found in this study, is lower than that reported in humans. In the human 1000 genome pilot phase project, population-scale CNV mapping with 185 individuals revealed a total of 22,025 deletions and 6000 additional SV [39]. Recently, the human 1000 genome project released phase III integrated SV data. A total of 330.3 Mb of CNV gains (duplications) and 350.2 Mb of CNV losses (deletions) were reported, with 3.63 Mb inversions, 5.6 kb small insertions and 1.54 Mb indels. The fact that, in cattle, we identified a lower proportion of the genome involved in SV may reflect the stringent filters, which we adopted, and the smaller number of total sequence reads in our data set, rather than species differences.

There is some overlap between our SV set and that reported in other bovine SV studies. Hou et al. [40] reported 3438 CNV regions (CNVR) that cover 146.9 Mb on the UMD 3.1 assembly from 630 cattle of 27 breeds using BovineHD BeadChip information, of which 1360 were unique to only one sample. Thirty-six percent of the identified variable sequence space were also reported in their previous study [41]. Compared to our SNP chip results, we found 1295 CNVR that covered 53.2 Mb and overlapped with the Hou et al. [41] set, from which 774 of the 2135 CNVR (33.4 Mb/243.9 Mb, ~ 13.7%) and 1023 of the 2833 CNVR (42 Mb/346.3 Mb, 12.13%) were also found in the Holstein and Jersey sets, respectively. We also found that about 36% of the CNVR reported by Hou et al. overlapped with our genome sequence set. Jiang et al. [42] reported 358 CNVR that covered 34.45 Mb of the 29 bovine autosomes using the BovineHD BeadChip on 96 Chinese Holstein cattle. Two hundred and eighty of the 358 (78.2%) CNVR that covered 21.33 Mb were also confirmed by our SNP chip results. Several comparative genomic hybridization array (array-CGH) based studies have reported CNV. Liu et al. [11] reported CNV that spanned 28.1 Mb of the genome from 90 animals using array-CGH and Kijas et al. [43] reported 51 CNV that spanned about 1.33 Mb of the genome from 10 animals. Since we did not use an array-CGH based method for SV detection, we did not compare our specific SV locations with those reported in these two latter studies.

Among the other sequence-based SV studies, Zhan et al. [44] reported 8596 SV that covered 6.28 Mb using Breakdancer and 1416 SV that covered 1.15 Mb using Pindel from one Holstein bull, and Bickhart et al. [45] detected 55.6 Mb of the bovine genome that encompassed SV from five individuals using a RD-based method. Our population SV calls (27.36 Mb in Holstein and 8.64 Mb in Jersey) represent a smaller proportion of the genome, although the sample size was larger in our study. As described in the Method section, this is likely the result of the very stringent pipeline used for SV detection and validation, which has the advantage that SV were called with lower false positive rates, but false negatives undoubtedly do occur.

The comparison between our PennCNV calls and large SV (larger than 5 kb) suggested a low overlapping rate (only 12 to 15%). This low percentage is mainly due to the different spectrums of detection: RP and SR methods are sensitive to small and medium-sized SV because of the limit set on insert size while SNP chip data only capture large SV. One potential better comparison would be to use an RD method such as CNVnator [46] and jointSLM [47] that target large SV events and then to compare them with SV from SNP chip data.

Another consideration is that SV detection from WGS data relies on differences with a reference genome, thus the quality of the bovine reference genome assembly is very important. Compared with the human genome, the quality of the bovine genome assembly is lower, which makes it more difficult to detect SV in the bovine genome than in the human genome, and perhaps resulting in a higher rate of false positive SV [48]. Furthermore, the mean coverage of most individuals in our study was not as high as for the human 1000 genome project (20 to 60×), thus limiting the power of the detection methods. With a higher coverage, both the accuracy and sensitivity can be improved and an additional strategy such as the read depth method could be used for SV analysis.

Our simulation results suggested that combining two methods (e.g. Breakdancer and Pindel, paired read mapping information and split read mapping information) can detect higher quality SV calls with less false positives. Although we aimed at mimicking real sequence data in the simulation by considering sequencing and alignment errors and repetitive regions, we do expect more false positives in the empirical than in the simulation data. To minimize miss-calls from low-complexity regions and poorly mapped regions, we also set a threshold in Pindel that allowed a maximum number of supporting reads (twice the genome coverage for deletions, insertions and inversions and four times for duplications) to report an SV event. In the end, pursuing validation in empirical data seemed a better strategy to define a SV set, than further refining simulations.

A potential limitation of our study is that we used alignment (BAM) files that were created with BWA [25], as provided to the 1000 Bull Genomes project. The project guidelines specify that reads are uniquely mapped and trimmed for base quality, which is likely to reduce the number of SV detected, especially in repetitive regions where unique positions are difficult to define. However, unfiltered raw reads may be associated with higher base and mapping error rates, which could lead to more false positives. Our primary aim was not to identify all possible putative SV, but rather to identify a subset of SV that have a high probability of being true SV.

When we tested the hypothesis that the number of SV spanning genes should be smaller in genes that are highly conserved across species, we found that eight of the 237 CEG were encompassed by SV of at least 50 bp. The Chi squared test results indicated that SV were less common in the regions of genes that are highly conserved across eukaryotes. One of the eight genes, *DENR* on BTA29, was completely covered by a deletion SV. We further looked into the individuals that harbor this deletion and found that they were all heterozygous deletions.

We also identified several gene families that were expanded in the bovine genome and were associated with SV. Within the *beta*-*defensin* gene family, nine of the 34 (26.5%) genes were spanned by different types of SV. In cluster B, both deletions and insertions were located within *DEFB122* and *DEFB125*, two genes that bear a closer similarity to each other than to any other *defensin* genes in the bovine genome [32]. Interestingly, the expression of *DEFB122* differs significantly between Norwegian Red and Holstein cattle; *DEFB125* was expressed in the mature bull epididymis and vas deferens, but was absent from immature male and female individuals [49]. In cluster D of the *beta*-*defensin* gene family, Bickhart et al. [45] showed that *DEFB* and *DEFB1* harboured SV and that the copy number of *DEFB1* varied between *Bos indicus* and *B. taurus* and among Angus, Holstein and Hereford breeds. We also identified SV in the *cathelicidin*, *PAG* and *PRP* gene families, with *CATHL2* from the *cathelicidin* family being entirely encompassed by an SV.

L1-mediated retrotransposons are associated with various forms of SV and with human genetic diseases [38], which suggests that they may be a major source of genetic structural variation and evolution [50]. In our study, we detected more deletions in the L1 regions than in the other non-exonic regions, whereas there was no significant difference for the three other SV. In general, our findings support the hypothesis that recent expansions of gene families derived from SV/CNV have provided another source of genetic variation during evolution.

## Conclusions

Using information from split reads and pair-end mapping, as well as stringent filtering of data from twice-sequenced animals and evidence of sire-son transmissions, we were able to identify a catalogue of higher confidence SV in two bovine breeds (Holstein and Jerseys). A large proportion of the SV were shared between these two breeds, which suggests that at least some SV are common across breeds. We found that SV were depleted in genes that are highly conserved across eukaryotes and enriched in gene families that are expanded in the bovine genome and L1 regions. Three immune-related genes, *DEFB*, *DEFB*1 and *CATHL2* were completely encompassed by SV, which confirms results from other studies. The set of SV described here could be useful for the identification of potential causative variants in QTL regions [21]. Furthermore, the incorporation of SV genotypes into genomic prediction may increase the accuracy of genome estimated breeding values for some traits and lead to additional genetic gain.

## Additional files

**Additional file 1: Figure S1**. Flowchart for simulation pipeline; **Figure S2**. Flowchart for population SV pipeline; **Figure S3**. Flowchart for validated SV pipeline; **Figure S4**. Venn diagram for validated SV set pipeline. The flowcharts represent the pipeline of simulating SV and detecting population and validated SV sets.

**Additional file 2: Table S1.**Sample animals with sequence coverage and SRA accession number. This file contains for each sample international ID, breed, sequencing coverage and SRA accession number.

**Additional file 3: Tables S2**. Summary statistics of Breakdancer (BD), Pindel (PD) and Overlap method in HOM and HET simulation sets; **Table S3**: Precision of each SV detection method for deletions, inversions and duplications in all regions and repetitive regions; **Table S4**: Average number of SV and SV covered regions (Mb) per sample in the Holstein and Jersey populations detected by Breakdancer and Pindel; **Table S5**: Number of SV in each whole-genome sequence of 28 twice-sequenced individuals and in TWICE_SEQ set. The data provided summarise the SV output from simulation, empirical population set and twice-sequenced validated set.

**Additional file 4: Table S6**. Results for the validated SV sets. This file contains results of three validated SV sets.

**Additional file 5: Table S7**. RefSeq genes that overlap with SV. This file contains the RefSeq genes that overlapped with the validated SV set and the length of the overlap and percentage covered for those genes.
