# Erratum to: Detection and validation of structural variations in bovine whole-genome sequence data

**DOI:** 10.1186/s12711-017-0305-6

**Published:** 2017-03-03

**Authors:** Long Chen, Amanda J. Chamberlain, Coralie M. Reich, Hans D. Daetwyler, Ben J. Hayes

**Affiliations:** 1 AgriBio, Centre for AgriBioscience, Biosciences Research, Department of Economic Development, Jobs, Transport and Resources, Bundoora, VIC Australia; 2 School of Applied Systems Biology, La Trobe University, Bundoora, VIC Australia

## Erratum to: Genet Sel Evol (2017) 49:13 DOI 10.1186/s12711-017-0286-5

After publication of this work [1], we noted that Acknowledgement section was incomplete and should be as follows:

**Acknowledgements**

The authors are very grateful to all members of the 1000 Bull Genomes Consortium for provision of data. This paper is part of the collection ‘ISAFG2015’ (6th International Symposium on Animal Functional Genomics, 27–29 July 2015, Piacenza, Italy). The publication of the papers in this collection was partly sponsored by OECD Co-operative Research Programme: Biological Resource Management for Sustainable Agricultural Systems (CRP). Ben J Hayes’s participation in ISAFG2015 was financed by the OECD Co-operative Research Programme. The opinions expressed and arguments employed in this paper are the sole responsibility of the author and do not necessarily reflect those of the OECD or of the governments of its member countries.
