# Supplementary material for: Detection and validation of structural variations in bovine whole-genome sequence data
Source: Genet Sel Evol. 2017 Jan 25;49:13. doi: 10.1186/s12711-017-0286-5 (PMC5267451; doi:10.1186/s12711-017-0286-5)
Supplement: Supplementary file 1 — Additional file 1: Figure S1. Flowchart for simulation pipeline; Figure S2. Flowchart for population SV pipeline; Figure S3. Flowchart for validated SV pipeline; Figure S4. Venn diagram for validated SV set pipeline. The flowcharts represent the pipeline of simulating SV and detecting population and validated SV sets. [file 12711_2017_286_MOESM1_ESM.docx]

## Supplementary Figure S1 - Flowchart for simulation pipeline

***Homozygous SVs***

***Heterozygous SVs***

BTA29 genome sequence from UMD 3.1

Insert simulated SVs

(50 for each type)

Insert simulated SVs

(100 for each type)

100 sets of rearranged BTA29

50 sets of rearranged BTA29

Simulated read with ~ 5x Coverage

Simulated read with ~ 10x Coverage

Combine reads from two different rearranged BTA29 and map simulated reads to reference

Map simulated reads to reference

50 Bam files ready for SV analysis

(10x coverage)

50 Bam files ready for SV analysis

(10x coverage)

For homozygous SVs, 100 randomly distributed deletions, inversions and duplications, respectively, were inserted into BTA29 to form a rearranged BTA29. 50 replicate sets of rearranged BTA29 were generated and, based on each rearranged chromosome, 50 replicates of short reads were then simulated with ~10 fold sequence coverage. For heterozygous SVs, both the number of SVs and coverage per SV were halved to 50 and 5, respectively. 100 rearranged BTA29 chromosomes were then generated and reads simulated from two different rearranged BTA29 chromosomes were pooled together to simulate animals heterozygous for the SVs

## Supplementary Figure S2 - Flowchart for population SV pipeline

Run Main program to detect SVs

This flowchart illustrates the pipeline for generating population SV calls for Holstein and Jersey populations. In the filtering step, for both Breakdancer and Pindel, only SVs that were detected in at least 2 animals and > 50 bp regions from genome gaps were included. A few filters were further applied to the raw output of Pindel by setting: minimum coverage as 1; minimum SV event size as 50; minimum supporting reads as 5; maximum supporting reads as twice as the genome coverage for deletions, inversions and insertions and as 4 times of the genome coverage for tandem duplications. The final confident SV sets were then overlapped from filtered Breakdancer and Pindel output set by each SV type, respectively.

Confident SV calls: overlapped SVs from Breakdancer and Pindel for each type of SV within population

Filtered Breakdancer output in bed format for each chromosome and each type of SV within breed

Filtered Pindel output in vcf format

Apply filters

Convert file format

Pindel raw output for each chromosome and each SV type within breed

Breakdancer raw output for each sample

Breakdancer configuration files for each sample

Pindel configuration file contains all samples

Estimate insertion size

1.Paired-end sequencing data (Aligned by BWA): Bam and bam index file

2. Reference genome UMD3.1

## Supplementary Figure S3 - Flowchart for validated SV sets pipeline

Filters applied here

Convert file format

Pindel output in vcf format for each individual

Pindel raw output

Run Main program to detect SVs

Breakdancer output in bed format for each individual

Breakdancer raw output

Estimate insertion size

Breakdancer configuration file

Pindel configuration file

1.Paired-end sequencing data (Aligned by BWA): Bam and bam index file

2. Reference genome UMD3.1

Confident SV calls: overlapped SVs from Breakdancer and Pindel for each individual

Validated SV set using twice-sequenced individuals:

Confident SVs from sequence 1

Validated SVs for each individual

Confident SVs from sequence 2

Validated SV set for twice-sequenced individuals by merging SV calls from all twice-sequenced individuals

Confident SVs from sire

Validated SVs in each sire-son pair

Validated SV sets using sire-son pairs:

Confident SVs from son

Validated SV set for sire-son pairs by merging SV calls from all sire-son pairs within population

This flowchart illustrate the pipeline for generating SV calls for each individual and validated SV sets. The filters applied here were the same as in the population calls except the minimum number of supporting animals. Based on the confident SV calls for each individual, two validated SV sets were generated:

1. Twice-sequenced validated SV set was further overlapped between two independent sequences, and then merged among all the 28 individuals;

2. Sire-son validated sets were further overlapped between sire-son pairs and then merged within each breed. For each SV set we merged SVs by each SV type (DEL,INS,INV,DUP) if any SVs are adjacent to each other, i.e. at least 1 bp overlapped.

This flowchart illustrates a brief pipeline for generating SV calls for each individual. Based on the final SVcalls three different sets were generated:1. Population SV sets were merged within breed and were only included when detected in at least 2 animals; 2.Twice-sequenced validated SV set was further overlapped between two independent sequence, and then merged among all the 28 individuals; 3. Parent-offspring validated sets were further overlapped between sire-son pairs and then merged within each breed. For each SV set we merge SVs by each SV type (DEL,INS,INV,DUP) if any SVs are adjacent to each other, i.e. at least 1 bp overlapped.

## Supplementary Figure S4 – Venn diagram for validated SV set pipeline

Sequence 2 (Son)

Sequence 1 (Sire)

Validated Structural Variations

Confident SVs from S1

Confident SVs from S2

Breakdancer

Pindel

Breakdancer

Pindel

For TWICE_SEQ set, sequence 1 and sequence 2 represent the two sequences for each individual; for FAM_HOL and FAM_JER set, sequence 1 represents the sequence from sire and sequence 2 is the sequence from son. We first generated the overlap SVs between Breakdancer and Pindel, and then overlapped those SVs between two sequences to get validated SVs.
