# Supplementary material for: Detection and validation of structural variations in bovine whole-genome sequence data
Source: Genet Sel Evol. 2017 Jan 25;49:13. doi: 10.1186/s12711-017-0286-5 (PMC5267451; doi:10.1186/s12711-017-0286-5)
Supplement: Supplementary file 3 — Additional file 3: Tables S2. Summary statistics of Breakdancer (BD), Pindel (PD) and Overlap method in HOM and HET simulation sets; Table S3: Precision of each SV detection method for deletions, inversions and duplications in all regions and repetitive regions; Table S4: Average number of SV and SV covered regions (Mb) per sample in the Holstein and Jersey populations detected by Breakdancer and Pindel; Table S5: Number of SV in each whole-genome sequence of 28 twice-sequenced individuals and in TWICE_SEQ set. The data provided summarise the SV output from simulation, empirical population set and twice-sequenced validated set. [file 12711_2017_286_MOESM3_ESM.docx]

## Supplementary Table S2 – Summary statistics of Breakdancer (BD), Pindel (PD) and Overlap method in HOM and HET simulation sets

|  | Homozygous | | Heterozygous | |
| --- | --- | --- | --- | --- |
| Program | True Positives | Total Calls | True Positives | Total Calls |
| BD_DEL | 81.86(3.31) | 83.05(3.82) | 76.25(3.66) | 80.58(4.11) |
| BD_INV | 94.73(1.79) | 96.60(4.60) | 86.52(2.07) | 96.39(2.55) |
| BD_DUP | 79.68(4.21) | 79.81(4.18) | 79.44(3.85) | 79.79(3.91) |
| PD_DEL | 75.61(5.07) | 76.44(4.96) | 39.87(4.83) | 40.15(4.79) |
| PD_INV | 68.23(6.00) | 69.80(5.87) | 58.06(4.80) | 58.85(4.83) |
| PD_DUP | 79.57(4.18) | 80.46(3.87) | 73.71(4.81) | 73.91(4.79) |
| OVERLAP_DEL | 61.93(4.75) | 61.93(4.75) | 31.63(4.49) | 31.63(4.49) |
| OVERLAP_INV | 66.64(5.72) | 66.76(5.70) | 57.06(4.86) | 57.13(4.87) |
| OVERLAP_DUP | 60.17(4.60) | 60.17(4.60) | 56.66(5.14) | 56.66(5.14) |

Numbers indicate the average count of calls from 50 simulations. The number in parentheses is the standard deviations of the counts. BD, Breakdancer. PD, Pindel. OVRLAP, overlap of SVs in Breakdancer and Pindel. DEL, deletions. INV, inversions. DUP, duplications.

## Supplementary Table S3 - Precision of each SV detection method for deletions, inversions and duplications in all regions and repetitive regions (BD=Breakdancer, PD=Pindel, OVERLAP=overlap of the two methods).

|  | **All regions** | **Repetitive regions** |
| --- | --- | --- |
| BD_DEL | 96.13% | 77.09% |
| BD_INV | 94.23% | 32.96% |
| BD_DUP | 99.72% | 91.74% |
| PD_DEL | 98.95% | 99.43% |
| PD_INV | 97.75% | 99.82% |
| PD_DUP | 98.98% | 90.78% |
| OVERLAP_DEL | 100.00% | 99.62% |
| OVERLAP_INV | 99.76% | 99.78% |
| OVERLAP_DUP | 99.99% | 99.46% |

## Supplementary Table S4 – Average number of SV and SV covered region (Mb)s in the Holstein and Jersey populations detected by Breakdancer and Pindel

|  | Breakdancer |  |  |  | Pindel |  |  |  |
| --- | --- | --- | --- | --- | --- | --- | --- | --- |
| Holstein | Deletions | Insertions | Inversion | Duplications | Deletions | Insertions | Inversion | Duplications |
| SV Count per sample | 4707.492 | 8389.357 | 1351.131 | 6825.730 | 1547.627 | 178.803 | 331.221 | 611.324 |
| SV covered region (MB) per sample | 5.500 | 1.380 | 4.666 | 2.624 | 0.923 | 0.012 | 0.518 | 0.327 |
| Jersey |  |  |  |  |  |  |  |  |
| SV count per sample | 1736.266 | 0.266 | 625.844 | 6100.953 | 983.516 | 72.781 | 68.188 | 223.156 |
| SV covered region (MB) per sample | 4.580 | 0.000 | 3.666 | 2.507 | 0.377 | 0.005 | 0.191 | 0.120 |

## Supplementary Table S5 – Number of SVs in each sequence of 28 twice sequenced individuals and in TWICE_SEQ set

|  | Sequence 1 | |  |  | Sequence 2 | |  |  | Overlap between two sequences | | | |
| --- | --- | --- | --- | --- | --- | --- | --- | --- | --- | --- | --- | --- |
| Animal ID | DEL | INS | INV | DUP | DEL | INS | INV | DUP | DEL | INS | INV | DUP |
| HOLCANM253 | 564 | 0 | 12 | 51 | 2287 | 0 | 48 | 142 | 257 | 0 | 8 | 21 |
| HOLCANM254 | 4005 | 553 | 33 | 79 | 3825 | 0 | 71 | 218 | 1112 | 0 | 47 | 102 |
| HOLDEUM255 | 4216 | 859 | 31 | 53 | 2855 | 636 | 27 | 34 | 2168 | 14 | 40 | 52 |
| HOLDEUM256 | 5529 | 405 | 32 | 27 | 3798 | 442 | 33 | 21 | 2730 | 31 | 45 | 25 |
| HOLDEUM257 | 4966 | 839 | 41 | 42 | 6064 | 773 | 41 | 42 | 3626 | 38 | 69 | 48 |
| HOLDEUM258 | 116 | 0 | 0 | 2 | 2985 | 455 | 40 | 72 | 2 | 0 | 0 | 3 |
| HOLDNKM259 | 4116 | 671 | 27 | 45 | 2751 | 643 | 16 | 30 | 2001 | 7 | 25 | 28 |
| HOLDNKM260 | 1605 | 197 | 9 | 17 | 6328 | 876 | 43 | 55 | 1305 | 4 | 11 | 21 |
| HOLDNKM261 | 2519 | 600 | 25 | 28 | 4491 | 696 | 33 | 36 | 1867 | 7 | 31 | 25 |
| HOLDNKM262 | 3458 | 448 | 26 | 43 | 4841 | 474 | 32 | 47 | 2537 | 42 | 37 | 62 |
| HOLDNKM263 | 3772 | 724 | 34 | 35 | 6722 | 643 | 36 | 40 | 2697 | 10 | 50 | 37 |
| HOLFRAM264 | 603 | 0 | 5 | 14 | 121 | 0 | 0 | 0 | 1 | 0 | 0 | 0 |
| HOLFRAM265 | 1189 | 0 | 29 | 125 | 3050 | 0 | 1981 | 168 | 573 | 0 | 36 | 86 |
| HOLFRAM266 | 2200 | 434 | 17 | 20 | 4491 | 748 | 33 | 38 | 1635 | 13 | 23 | 18 |
| HOLFRAM267 | 1504 | 0 | 38 | 149 | 2745 | 145 | 249 | 171 | 999 | 0 | 41 | 93 |
| HOLFRAM268 | 931 | 0 | 21 | 115 | 29 | 0 | 1 | 18 | 27 | 0 | 0 | 26 |
| HOLGBRM269 | 164 | 0 | 0 | 10 | 1337 | 0 | 23 | 77 | 19 | 0 | 0 | 4 |
| HOLNLDM270 | 2738 | 538 | 25 | 18 | 4250 | 660 | 33 | 28 | 2069 | 8 | 31 | 23 |
| HOLNLDM271 | 1392 | 0 | 37 | 145 | 1223 | 1 | 22 | 146 | 384 | 0 | 15 | 79 |
| HOLNLDM272 | 5337 | 874 | 34 | 44 | 3447 | 684 | 23 | 31 | 2520 | 17 | 32 | 43 |
| HOLNLDM273 | 4217 | 776 | 29 | 38 | 2484 | 638 | 20 | 25 | 1913 | 3 | 29 | 31 |
| HOLNLDM274 | 2990 | 711 | 45 | 33 | 2407 | 816 | 40 | 26 | 1585 | 23 | 42 | 41 |
| HOLSWEM275 | 4886 | 782 | 36 | 47 | 3942 | 877 | 37 | 40 | 2882 | 18 | 50 | 49 |
| HOLUSAM276 | 665 | 0 | 2 | 11 | 2394 | 496 | 16 | 25 | 132 | 0 | 0 | 5 |
| HOLUSAM277 | 5195 | 913 | 46 | 45 | 3133 | 766 | 36 | 21 | 2398 | 10 | 54 | 22 |
| HOLUSAM278 | 5132 | 696 | 34 | 38 | 4249 | 772 | 34 | 30 | 3067 | 36 | 54 | 32 |
| HOLUSAM279 | 4178 | 719 | 36 | 45 | 3659 | 825 | 37 | 29 | 2581 | 13 | 54 | 28 |
| HOLUSAM280 | 4581 | 771 | 35 | 54 | 2927 | 745 | 27 | 38 | 2200 | 11 | 36 | 49 |
| Total | 82768 | 12510 | 739 | 1373 | 92835 | 13811 | 3032 | 1648 | 45287 | 305 | 860 | 1053 |
